# Predictive Response to Immunotherapy Score: A Useful Tool for Identifying Eligible Patients for Allergen Immunotherapy

**DOI:** 10.3390/biomedicines10050971

**Published:** 2022-04-22

**Authors:** Ilaria Mormile, Francescopaolo Granata, Aikaterini Detoraki, Daniela Pacella, Francesca Della Casa, Felicia De Rosa, Antonio Romano, Amato de Paulis, Francesca Wanda Rossi

**Affiliations:** 1 Department of Translational Medical Sciences, University of Naples Federico II, 80131 Naples, Italy; ilariamormile@virgilio.it (I.M.); francesca.dellacasa4@gmail.com (F.D.C.); felicia.derosa@libero.it (F.D.R.); depaulis@unina.it (A.d.P.); francescawanda.rossi@unina.it (F.W.R.); 2 Department of Internal Medicine, Clinical Immunology, Clinical Pathology, and Infectious Diseases, Azienda Ospedaliera Universitaria Federico II, 80131 Naples, Italy; caterina.detoraki@gmail.com; 3 Department of Public Health, University of Naples Federico II, 80131 Naples, Italy; daniela.pacella@unina.it; 4 Department of Neurosciences, Reproductive and Odontostomatological Sciences, Maxillofacial Surgery Unit, University of Naples Federico II, 80131 Naples, Italy; romano.antonio1972@gmail.com; 5 Center for Basic and Clinical Immunology Research (CISI), WAO Center of Excellence, University of Naples Federico II, 80131 Naples, Italy

**Keywords:** allergic rhinitis, allergen immunotherapy, bronchial asthma, component-resolved diagnosis, sublingual immunotherapy

## Abstract

A specific predictive tool of allergen immunotherapy (AIT) outcome has not been identified yet. This study aims to evaluate the efficacy of a disease score referred to as Predictive Response to Immunotherapy Score (PRIS) to predict the response to AIT and identify eligible patients. A total of 110 patients diagnosed with allergic rhinitis with or without concomitant asthma were enrolled in this study. Before beginning sublingual immunotherapy (SLIT), patients were evaluated by analyzing clinical and laboratory parameters. A specific rating was assigned to each parameter to be combined in a total score named PRIS. At baseline (T0) and follow-up [after 12 (T12) and 24 months (T24) of SLIT], a Visual Analogue Scale (VAS) was used to calculate a mean symptom score (MSS). Finally, the percentage variation between the MSS at T0 and at T12 [ΔMSS-12(%)] and T24 [ΔMSS-24 (%)] was measured. We observed a significant improvement of symptoms at T12 and T24 compared to T0 in all groups undergoing SLIT. PRIS was effective in predicting ΔMSS-24 (%) in patients treated with single-allergen SLIT. In addition, PRIS was effective in predicting ΔMSS-24 (%) in both patients with only rhinitis and with concomitant asthma. PRIS assessment can represent a useful tool to individuate potential responders before SLIT prescription.

## 1. Introduction

Allergic rhinitis and bronchial asthma are widespread diseases that can impact social life, school learning, and work productivity when poorly controlled by conventional therapy [1]. In addition, allergic rhinitis is considered one of the major risk factors for asthma, as up to 40% of patients with allergic rhinitis have or will go on to develop it [2]. Allergen immunotherapy (AIT) is the only disease-modifying treatment for allergic diseases, as it can prevent both the onset of new allergic sensitizations and disease progression [3]. AIT should be considered in those subjects with inadequate response or adverse effects to conventional medications such as antihistamines, topical intranasal antihistamines, and intranasal corticosteroid sprays [4,5,6]. Two routes of administration of AIT, subcutaneous (SCIT) or sublingual (SLIT), are currently used in clinical practice and have shown good efficacy in the treatment of allergic rhinitis and bronchial asthma [7]. International guidelines recommend that maintenance therapy for both SCIT and SLIT should be continued for at least three years [4,6,7]. Hence, AIT is a long-lasting and expensive treatment, especially if the patient is being treated for more than a few allergens. In addition, in clinical studies, it has been frequently observed that a percentage of patients undergoing AIT do not have significantly beneficial effects [8]. Indeed, the efficacy of AIT ranges between 60% and 90% [9].

Several studies have tried to identify some biomarkers able to predict AIT response through the years. For instance, the assessment of serum-specific IgE [10] and the specific and total IgE ratio has been proposed as a biomarker of AIT efficacy [11]. Other studies suggested a possible correlation between some subtypes of IgG (IgG1, sIgG4) and clinical outcomes [12,13]. Finally, changes in cytokine pattern (e.g., IL-4, IL-13, IL-10) have been associated with AIT response [9,14,15]. However, there is no consensus on the use of these markers in the clinical routine.

The diagnostic approach to allergic disease has significantly been improved by the Component-Resolved Diagnosis (CRD), which provides information about patients’ sensitization at the molecular component level by integrating the Skin Prick Test (SPT) and the specific IgE assay with extractive allergen results. Indeed, CRD can increase awareness about the major allergen sensitization and help avoid the administration of AIT for irrelevant allergens, improving its clinical efficacy and cost effectiveness [16,17]. However, the studies which tried to establish a direct link between CRD and AIT outcomes have shown conflicting results.

This study aims to develop and validate a disease score, referred to as Predictive Response to Immunotherapy Score (PRIS), combining clinical features and laboratory results to predict the likelihood of clinical improvement during AIT and identify eligible patients.

## 2. Materials and Methods

### 2.1. Patients

Defining the primary outcome as the relationship between the total PRIS score and the ΔMSS, a minimum sample size of 85 patients achieves 80% power assuming a medium effect size (d = 0.3; [18]). A two-tailed test on Pearson’s correlation was considered with a significance level α = 0.05. Therefore, 110 patients (68 males and 42 females) diagnosed with allergic rhinitis with or without concomitant asthma at the Division of Allergy and Clinical Immunology of the University of Naples Federico II, Naples, Italy, were enrolled in this prospective cohort single-center study. All the patients presented a history of symptoms related to allergen exposure (rhinitis and/or asthma), documented positive SPT for pollen and/or perennial allergens, and allergen-specific IgE test. Spirometry was used to diagnose asthma [19].

We included both monosensitized and polysensitized patients with uncontrolled allergies despite optimal pharmacotherapy. Patients under six years of age were excluded. We also excluded patients with asthma not adequately controlled by pharmacotherapy [20,21,22] as assessed by Asthma Control Test (ACT) [23,24,25]. Finally, we excluded patients with nasal polyposis diagnosed by nasal endoscopy.

Demographic and clinical data were collected from patient medical charts and diaries. Data were available for all 110 patients.

Rhinitis and asthma symptoms were singularly measured using a Visual Analogue Scale (VAS) at baseline (T0) and after 12 (T12) and 24 months (T24) of SLIT treatment. In particular, patients were asked to place a mark on a 10 cm line for rating the severity and frequency of each symptom [26,27]. The symptoms evaluated for allergic rhinitis were sneezing, nasal congestion, rhinorrhea, and nasal, throat, eyes, and ears itching, while chest tightness, breathlessness, wheezing, and coughing were assessed for bronchial asthma. The VAS was anchored at 0 with “no symptoms” and 10 with “very severe symptoms”. The VAS also included the assessment of the frequency of symptoms (0 with “no symptoms in the last 30 days” and at 10 with “I have experienced symptoms every day in the past 30 days”). In addition, we instructed patients to record monthly in a diary their symptoms, the number of asthma exacerbations, and on-demand therapy [28,29,30]. When the patients were visited, they were asked to complete the VAS by checking their diaries. This helped patients take note of their clinical conditions both during and out of season. In particular, when the VAS was administrated for evaluating the asthma exacerbation, 0 corresponded to no exacerbation while 10 implied frequent exacerbations. All the patients enrolled were switched to the same on-demand therapy with second-generation oral antihistamines and intranasal corticosteroids. Patients with asthma were treated using inhaled corticosteroids (ICS) and long-acting β-agonists (LABAs) as the controller and the quick relief therapy. The patients were instructed to record monthly on the provided diary the use of on-demand therapy. Subsequently, when the patients were visited, their perception of on-demand therapy use was evaluated by the VAS (10 implied the highest medication use, while 0 corresponded to no medication use). The mark was then measured in millimeters for all the items explored to provide the VAS score and normalized to 100. For each patient, we assessed the mean symptom score (MSS) based on VAS results at T0 (MSS-0), T12 (MSS-12), and T24 (MSS-24). As efficacy index of SLIT, we calculated the percentage difference between the MSS-0 and MSS-12 [ΔMSS-12(%) = (MSS-0–MSS-12)/MSS-0*100], and between MSS-0 and MSS-24 [ΔMSS-24(%) = (MSS-0–MSS-24)/MSS-0*100]. Based on the ΔMSS-12(%) and ΔMSS-24(%) results, patients’ SLIT outcome was stratified into quartiles (first quartile = ΔMSS ≥ 75% = very high symptom improvement; second quartile = 50% ≤ ΔMSS < 75% = high symptom improvement, third quartile = 25% ≤ ΔMSS < 50% = mild symptom improvement, fourth quartile = ΔMSS < 25% = low symptom improvement, Table 1).

All procedures performed in this study were in accordance with the ethical standards of the study center and with the 1964 Helsinki Declaration and its later amendments or comparable ethical standards. All the subjects enrolled gave informed consent to participate in the study.

### 2.2. Predictive Response to Immunotherapy Score (PRIS)

We tried to develop a specific disease index for predicting SLIT efficacy so that patients could choose whether to undergo SLIT based on their chance of success. We identified eight parameters that might contribute to SLIT responsiveness. Each parameter was assigned a score range, and three to five groups were established (Table 2). The parameters were chosen based on clinical practice, literature review, and previous work evaluating AIT responsiveness and possible predictive factors. The parameters included age, clinical features, disease onset, number of allergen sensitizations, presence of symptoms following exposure to the allergen(s) to which the patient is sensitized, specific IgE/total IgE ratio, IgE level for CRD, and allergen dominance (Table 2).

Total PRIS was calculated for each patient when he/she completed the diagnostic evaluation. Each patient was informed about his/her PRIS value and spontaneously decided to undergo SLIT treatment and be enrolled in this study. PRIS value could potentially range from 20 to 100. Therefore, PRIS stratification in quartiles would be as follows: first quartile = PRIS ≥ 80; second quartile = 80 > PRIS ≥ 60; third quartile = 60 > PRIS ≥ 40; fourth quartile = PRIS < 40. However, the PRIS values of patients enrolled in this study ranged from 41 to 93. Therefore, we had no patients in the fourth quartile.

### 2.3. Skin Prick Test

SPT was performed on the forearms of all enrolled subjects to confirm the diagnosis of a suspected type I allergy and identify the sensitization type. We used specific inhalant allergen extracts (Gramineae grass pollen (Gramineae mix/Phleum Pratense/Cynodon Dactilon), ambrosia, mugwort, wall pellitory (Parietaria Judaica/Parietaria Officinalis), olive pollen (Olea Europea), cypress pollen (Cupressaceae), birch, cat, dog, house dust mite (Dermatophagoides farina/Dermatophagoides pteronissinus), molds (Alternaria Alternata/Aspergillus/Cladosporium), a histamine positive control, and normal saline as a negative control. The test was interpreted after 15–20 min of application, with a positive result defined as a wheal ≥3 mm diameter.

### 2.4. In Vitro Tests

Total IgE and specific IgE Assay (ImmunoCAP 250; Phadia, Sweden) were performed in patients with positive SPT to evaluate the major inhalant allergen. The level of awareness towards the main inhalant allergen was increased using the CRD. We evaluated IgE antibodies to Phl p1 (Timothy grass), Phl p5 (Timothy grass), Bet v1 and Bet v2 (Betula verrucose), Amb a1 (Ambrosia), Art v1 (Mugwort), Par j2 (Wall pellitory), Ole e1 (Olea europea), Cup a1 (Cupressus arizonica), Fel d1 (cat), Can f1 (dog), Der p1 (House dust mite), Der p2 (House dust mite), and Alt a1 (Alternaria alternata). IgE levels were considered positive at the level ≥0.35 kUA/l. Patients with IgE antibodies to Bet v2 were excluded to rule out profilin allergy [31,32,33].

### 2.5. Immunotherapy

SLIT was performed using allergen medicines currently authorized and marketed in Italy (Oralvac Plus^®^/Allergy Therapeutics; Sulgen^®^/Roxall-Aristegui; SlitONE Ultra^®^, Grazax^®^, Accarizax^®^/ALK Abellò; Lais^®^/Lofarma). The allergen(s) used for immunotherapy (dominant allergen(s)) had to be clinically relevant to the patient’s clinical history, and it was identified according to the result of SPT and specific IgE assay. In detail, a difference of wheal diameter ≥5 mm compared to the other allergen tested at SPT and a difference of half a logarithm of the IgE level for a specific allergen compared to the other allergens was required to identify the dominant allergen(s). When applicable, the awareness of the major allergens was increased using CRD. Patients with one dominant allergen underwent a single-allergen SLIT; those with two dominant allergens underwent a two-allergen SLIT (Table 3). The evaluating physicians performed the first SLIT administration, then the patients were carefully instructed about the self-administration, and written instructions were provided to follow administration protocol.

### 2.6. Data Analysis

Data were summarized by descriptive analysis. Means and SD were calculated for continuous variables, while absolute values and frequency (percentage) were calculated for categorical variables. The assessment of the significance of the results obtained was performed with repeated-measures 1-way ANOVA with “MSS” (MSS-0, MSS-12, and MSS-24) as a within-subject factor. To test the predictive value of PRIS on ΔMSS-24(%) as well as of the PRIS parameters we used linear regression analysis. Analysis of dependent variable ΔMSS-24 was performed with independent 1-way ANOVA considering the stratification of patients according to the PRIS value as a between-subject independent variable (PRIS ≥ 80; 80 > PRIS ≥ 60; 60 > PRIS ≥ 40; PRIS < 40). The level of significance was set at α = 0.05.

## 3. Results

### 3.1. Demographic Data

A total of 110 patients, 68 males (61.8%) and 42 females (38.1%), were enrolled in this study. The cohort was White-Caucasian. The average age at enrollment was 24.87 ± 10.80 years (6–63). During AIT, 12 patients (10.9%) dropped and were excluded from the overall assessment which was performed only on the 98 patients who completed the 24-month immunotherapy. Therefore, the evaluation of a total of 98 patients who completed the 24-month SLIT treatment were included in the T12 and T24 evaluations. All patients enrolled were affected by allergic rhinitis (n = 98; 100%), and 49 out of 98 (50%) presented with concomitant allergic asthma.

### 3.2. Evaluation of Sublingual Immunotherapy Efficacy

Of the 98 total patients, 66 (67.34%) patients underwent SLIT for a single allergen and 32 (32.65%) underwent SLIT for two allergens (Table 3). Each patient received the maximum tolerated dose, per the manufacturers’ recommendations. SLIT was well tolerated, and no discontinuation due to severe adverse drug effects was registered.

**Figure 1:**
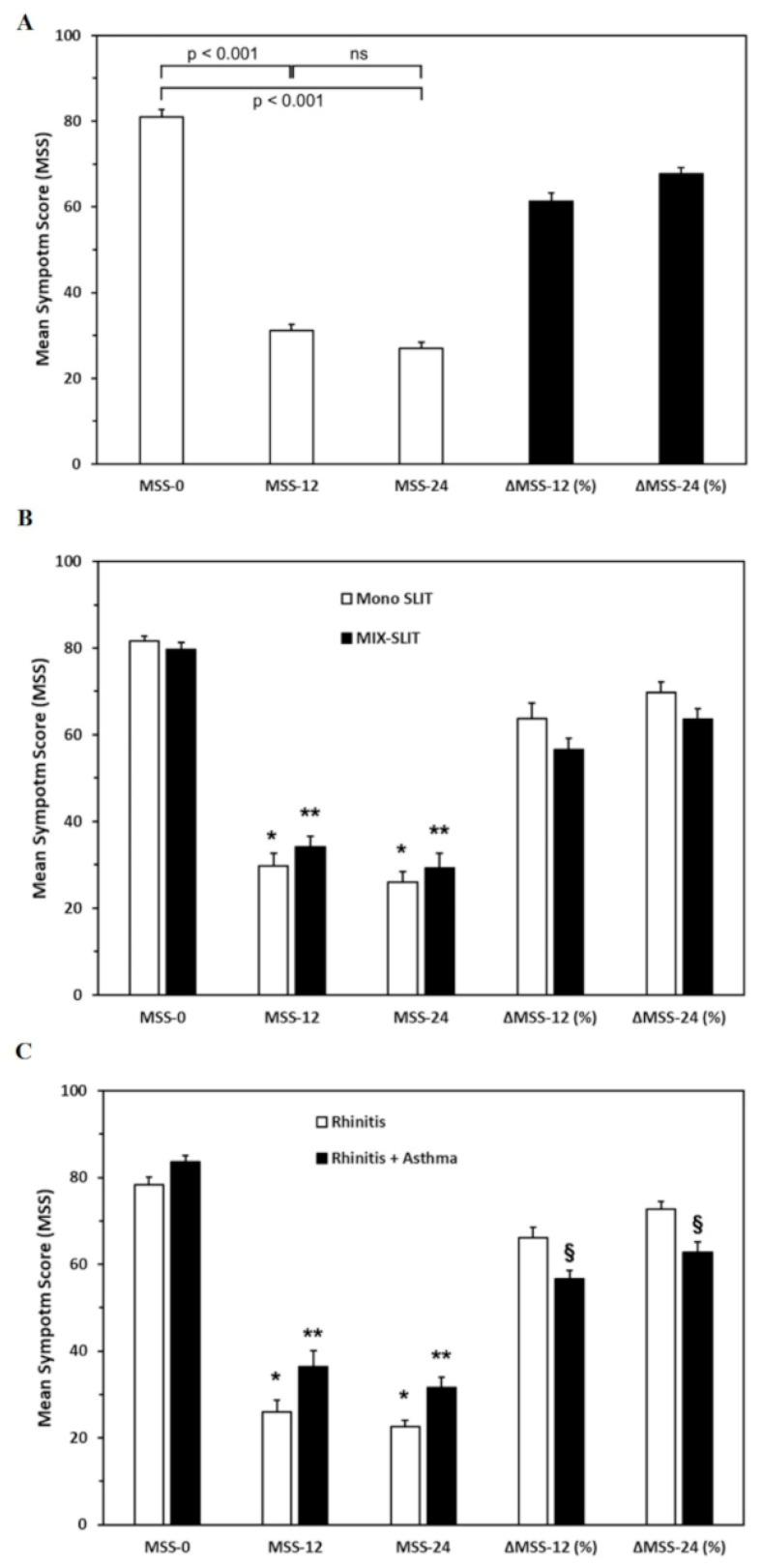
Sublingual immunotherapy (SLIT) efficacy assessment in the whole cohort (**A**), in patients treated with single-allergen (Mono SLIT) and two-allergen SLIT (MIX-SLIT) (**B**), and with allergic rhinitis and concomitant asthma (**C**). MSS-0: mean symptom score at T0; MSS-12: mean symptom score at T12; MSS-24: mean symptom score at T24; ΔMSS-12 (%): percentage difference between the MSS-0 and MSS-12; ΔMSS-24 (%): percentage difference between MSS-0 and MSS-24; ns: not significant; *,**: *p* < 0.001; §: *p* < 0.05.

Patients experienced a significant improvement in symptoms at T12 (mean MSS-12 = 31.11 ± 16.88) and T24 (mean MSS-24 = 27.07 ± 15.01) compared to T0 (mean MSS-0 = 80.97 ± 8.24). Indeed, ANOVA conducted on MSS revealed a significant difference between MSS-0 and MSS-12 (*p* < 0.001) and MSS-0 and MSS-24 (*p* < 0.001) (Figure 1A). Although an additional symptom improvement was recorded at T24, no significant difference was observable between MSS-12 and MSS-24 (*p* = 0.07). Accordingly, after 12 or 24 months of SLIT, the clinical improvement assessed by ΔMSS-12(%) and ΔMSS-24(%) was 61.35% and 67.71%, respectively.

To evaluate whether the number of allergens administered may affect SLIT efficacy, we compared patients undergoing single-allergen SLIT (Mono SLIT) with patients undergoing two-allergen SLIT (MIX-SLIT). Both patient groups showed a significant improvement of symptoms at T12 (Mono SLIT-MSS-12 = 29.68 ± 17.59; MIX-SLIT-MSS-12 = 34.06 ± 15.15) and T24 (Mono SLIT-MSS-24 = 26.00 ± 14.91; MIX-SLIT-MSS-24 = 29.28 ± 12.05) as compared to T0 (Mono SLIT-MSS-0 = 81.55 ± 7.75; MIX-SLIT-MSS-0 = 79.75 ± 9.18) (Figure 1B). In addition, no significant difference was found when ANOVA was conducted by comparing ΔMSS-12(%) and ΔMSS-24(%) in patients treated with a single-allergen SLIT and patients treated with a two-allergen SLIT (*p* = 0.11 and *p* = 0.07, respectively). These results indicate that the efficacy is comparable when one or two allergens are used for SLIT.

Next, we compared SLIT efficacy between patients with only rhinitis and rhinitis associated with asthma. Figure 1C illustrates that both patient groups showed a significant improvement in symptoms at T12 (rhinitis-MSS-12 = 25.90 ± 13.95; rhinitis+asthma-MSS-12 = 36.33 ± 18.05) and T24 (rhinitis-MSS-24 = 22.61 ± 11.71; rhinitis+asthma-MSS-24 = 31.53 ± 14.90) as compared to T0 (rhinitis-MSS-0 = 78.33 ± 8.55; rhinitis+asthma-MSS-0 = 83.60 ± 7.07). When ANOVA was conducted on ΔMSS, values revealed that ΔMSS-12(%) and ΔMSS-24(%) were significantly higher in patients with only rhinitis compared to patients with rhinitis and concomitant asthma (*p* < 0.05) (Figure 1C). These results indicate that SLIT was effective in both patients with allergic rhinitis and concomitant asthma. However, they also suggest that patients affected only by rhinitis can experience a better response to SLIT compared to patients with associated asthma.

### 3.3. Validation of the Predictive Response to Immunotherapy Score (PRIS)

**Figure 2:**
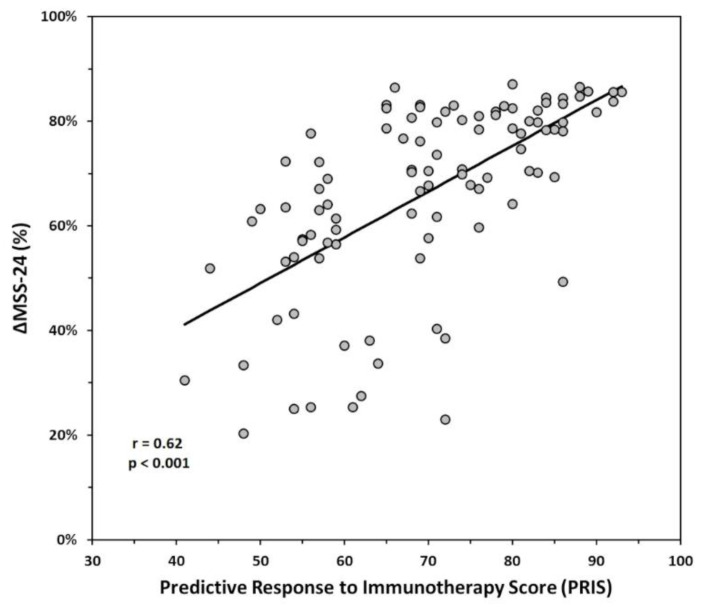
Correlation analysis revealed a significant direct correlation between the Predictive Response to Immunotherapy Score (PRIS) and ΔMSS-24 (*p* < 0.001).

Linear regression analysis was used to test the predictive value of PRIS on our efficacy index of SLIT [ΔMSS-24 (%)]. Figure 2 shows that overall PRIS significantly predicted ΔMSS-24 (%) (R = 0.622; F (1,97) = 60.810; *p* < 0.001).

**Figure 3:**
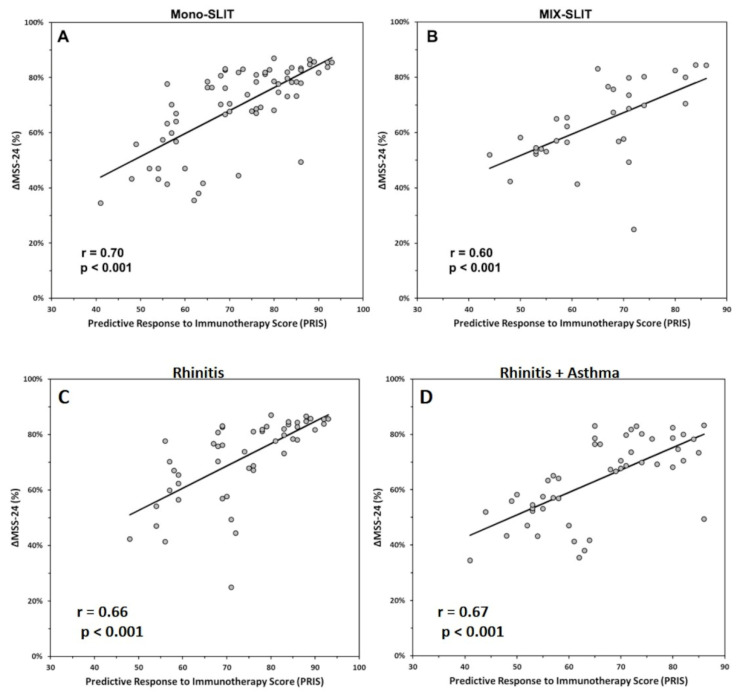
Correlation analysis revealed a significant direct correlation between the Predictive Response to Immunotherapy Score (PRIS) and ΔMSS-24 for both patients treated with single-allergen (**A**) and multiple-allergen (**B**) sublingual immunotherapy (SLIT) (*p* < 0.001), and between PRIS and ΔMSS-24 for both patients with only rhinitis (**C**) and with both asthma and rhinitis (**D**) (*p* < 0.001).

In addition, regression analysis verified that PRIS significantly predicted ΔMSS-24 (%) in patients treated with a single-allergen SLIT (Mono-SLIT: R = 0.708; F (1.65) = 64.453; *p* < 0.001; Figure 3A) as well as in patients treated with a two-allergen SLIT (MIX-SLIT: R = 0.599; F (1.31) = 16.833; *p* < 0.001; Figure 3B), suggesting that PRIS has the same efficacy in predicting SLIT outcome when one or two allergens are used for SLIT.

Furthermore, regression analysis also showed that PRIS significantly predicted ΔMSS-24 (%) in both patients with only rhinitis (R = 0.660; F (1.48) = 36.313; *p* < 0.001; Figure 3C) and in patients with rhinitis associated with asthma (R = 0.674; F (1.48) = 39.207; *p* < 0.001; Figure 3D), suggesting that PRIS is as effective as in predicting SLIT outcome in both patients with rhinitis and with concomitant asthma. Together these results indicate that PRIS can be used to predict the efficacy of SLIT independent of the number of allergens used with SLIT and the patient’s clinical condition.

Finally, in order to check that all parameters that compose the PRIS score contribute to the prediction of the outcome, linear regression analysis was also used to test the association of all individual PRIS components with ΔMSS-24 (%). As shown in Table 4, all PRIS parameters are significant predictors for our outcome, and the parameters’ score categories (assumed in the model on an ordinal scale) adequately reflect the difference progression in comparison with the references.

### 3.4. Stratification in Quartiles

We stratified patients into quartiles to gain insights into the relationship between immunotherapy efficacy and PRIS. Patients were first stratified in quartiles based on ΔMSS (%) to identify patients who had a better clinical response than those with a poor response to SLIT (Table 1). Overall, the vast majority of patients obtained a significant symptom improvement (very high or high) after 12-(72 out of 98 patients; 77.56%) and 24-month SLIT (82 out of 98 patients; 81.64%).

**Figure 4:**
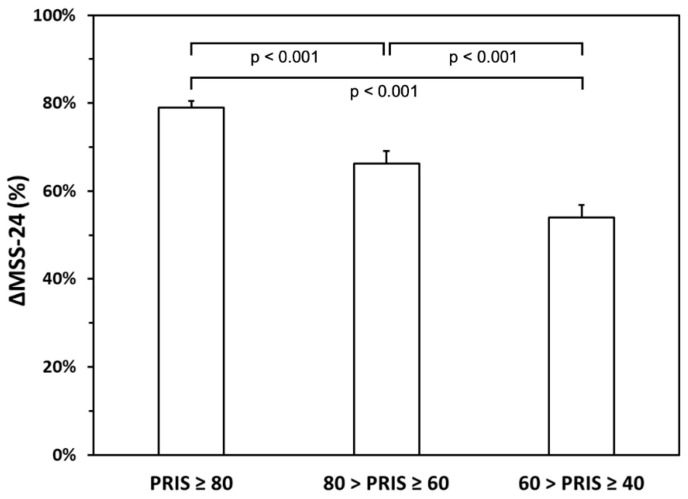
Stratification of patients in three groups according to the Predictive Response to Immunotherapy Score (PRIS) results and their ΔMSS-24. A significant difference was found between the three groups (F (2.97) = 16.32; *p* < 0.001).

We then stratified patients in quartiles based on PRIS values (PRIS ≥ 80; 80 > PRIS ≥ 60; 60 > PRIS ≥ 40) to identify patients who had more chances to obtain a significant response to SLIT. ANOVA conducted on ΔMSS-24 (%) revealed a significant difference between the three groups (F (2.97) = 16.32; *p* < 0.001). Post hoc comparisons revealed a significant higher value of ΔMSS-24 (%) for PRIS ≥ 80 (mean ± SD 78.91 ± 8.16) than 80 > PRIS ≥ 60 (post hoc *p* < 0.001; mean ± SD 66.25 ± 18.31) and 60 > PRIS ≥ 40 (post hoc *p* < 0.001; mean ± SD 54.02 ± 15.16) (Figure 4). In addition, ΔMSS-24 (%) for 80 > PRIS ≥ 60 (mean ± SD 66.25 ± 18.31) was significantly higher than 60 > PRIS ≥ 40 (post hoc *p* < 0.001; mean ± SD 54.02 ± 15.16). These results indicate that patients with a higher PRIS value have more chances to obtain a higher ΔMSS-24 (%).

**Figure 5:**
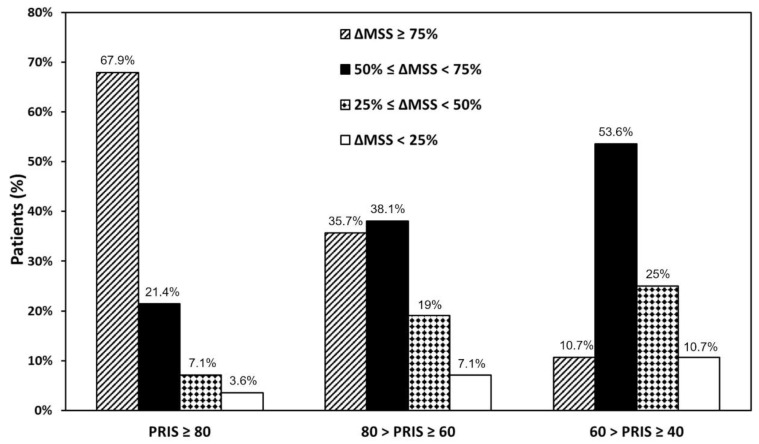
Stratification of patients in three groups according to the Predictive Response to Immunotherapy Score (PRIS) results and their ΔMSS-24. Most patients with a PRIS ≥ 80 experienced a very high improvement, whereas patients with 60 > PRIS ≥ 40 mostly experienced a high improvement. Patients with 80 > PRIS ≥ 60 were homogeneously distributed in ΔMSS-24 (%) quartiles.

Finally, we categorized patients by matching quartile stratification based on ΔMSS-24 (%) with quartile stratification based on PRIS. Figure 5 shows that most patients with a PRIS ≥ 80 experienced a very high improvement, whereas patients with 60 > PRIS ≥ 40 mostly experienced a high improvement. Patients with 80 > PRIS ≥ 60 were homogeneously distributed in ΔMSS-24 (%) quartiles. These data strongly suggest that PRIS can effectively predict the clinical response that patients may expect from SLIT.

## 4. Discussion

AIT is the only disease-modifying and potentially resolving treatment available for patients with IgE-mediated allergic diseases, and its efficacy has been proven with a high degree of evidence [34,35,36]. However, one of the major problems of AIT management in clinical practice is that current guidelines give no clear indication about the algorithm to be used for choosing patients eligible for this treatment [37,38,39,40]. This could be one of the reasons for the low patient compliance with AIT reported in many clinical studies [41,42,43]. The introduction of personalized medicine, envisioned as a patient-tailored diagnostic and therapeutic approach, is currently influencing all fields of medicine; therefore, dedicated tools for identifying patients eligible for AIT are strongly needed [13].

According to current guidelines, AIT is indicated in patients with allergic rhinitis, with or without co-existing asthma [37,38,39,40]. Identification of the allergen(s) driving symptoms is the first level of patient stratification to ensure that the correct allergen solution is used for AIT. However, this treatment is preferentially used in patients with few sensitizations or polysensitized patients with one to three dominant allergens in clinical practice. In addition, to take advantage of AIT long-term effects, younger patients with few allergic diseases and a recent onset of allergic rhinitis are preferred for AIT. Previous studies have tried to correlate AIT response with a single marker. For instance, changes in cytokine pattern, such as an increase in Th2-dependent cytokines IL-4 and IL-13, have been associated with AIT response [9]. In addition, IL-10 mRNA levels have been suggested to be predictive of clinical responses to AIT [14]. IL-10 producing regulatory B- and T-cells specific for allergens were reported to increase during AIT or following the natural allergen exposure [15]. However, no cytokine has been clearly established as a marker for AIT efficacy to be used in the clinical routine. In addition, the specific and total IgE ratio has been formerly proposed as a biomarker of AIT efficacy [11,44]. A study by Di Lorenzo et al. [11] analyzing 279 monosensitized patients treated with both SCIT and SLIT immunotherapy found that specific IgE/total IgE ratio >16.2 (i.e., specific IgE/total IgE ratio × 100) was associated with an effective response to AIT. On the contrary, a randomized, double-blind, placebo-controlled clinical trial by Fujimura et al. [44] reported that patients with specific IgE/total IgE ratio <0.19 achieved better AIT outcomes. Other authors have suggested considering allergen-specific IgE level rather than the specific IgE/total IgE ratio, describing higher specific IgE levels in AIT responders than in non-responder adults [45] and children with allergic rhinitis [46]. The same research group has proposed a cut-off value of allergen-specific IgE levels (>9.74 kUA/L) that could predict a successful response to AIT [10]. Nonetheless, these observations were based on a small number of studied patients, with a consistent discrepancy between the sample size of the responders and non-responders, which should be considered a limitation of these results [9]. We chose to use the specific IgE/total IgE ratio rather than allergen-specific IgE level, because we would need a normalized specificity index that could be easily stratified into categories. In addition, the linear regression analysis of PRIS parameters (Table 4) shows that the ratio was a significant PRIS predictor (*p* < 0.001). CRD can help to differentiate patients with genuine sensitization from those with cross-reactive sensitization to other allergen sources [1,17,47]. This could help avoid administering irrelevant allergens in AIT, improving its clinical efficacy and cost effectiveness [1,17,47]. A pilot study by di Coste et al. [1] including 36 children with allergic rhinoconjunctivitis monosensitized to grass pollen aimed to evaluate the correlation between the sensitization to different molecular Phleum pratense (Phl p) allergens and clinical efficacy of SLIT. The authors performed serum analysis of specific IgE to Phl p 1, 2, 4, 5, 6, 7, 11, and 12, and showed that SLIT was effective irrespective of the patients’ baseline sensitization to either single or multiple grass pollen allergens [1]. However, a direct correlation between IgE sensitization for other major allergens detected at the molecular component level and AIT outcome has currently not been found. Finally, other potential biomarkers that have been suggested for the assessment of AIT efficacy are the assessment of IgG1 and IgG4 levels [12] or the basophil activation test [48,49], but they did not show real reliability, and there is no consensus in their usage in patients undergoing SLIT [50]. Indeed, numerous studies indicate IgG1 and IgG4 levels increase during SLIT, but they may reflect compliance instead of clinical efficacy [12,50].

To approach the heterogeneity of allergic patients, we developed a multi-parameter score, namely PRIS, potentially able to predict AIT effectiveness and identify eligible patients. PRIS includes clinical and laboratory parameters (Table 2) chosen based on clinical practice, literature review, and previous work evaluating AIT responsiveness and possible predictive factors [1,10,11,12,13,44,46,48,49,51,52,53]. We rated each PRIS parameter to reach a maximum of 100 points to mimic the odds of achieving a clinical improvement, thereby making it easy to be used by clinicians and intelligible by the patients. We included age, number of allergic diseases, and disease onset as clinical parameters. In addition, we tried to increase the awareness of the major allergen(s) responsible for clinical symptoms by including in the PRIS the evaluation of the occurrence of symptoms following the exposure to one or more allergens [51]. As diagnostic parameters, we started by evaluating the sensitization profiles of patients, and we included the number of sensitizations, CRD, and the specific/total IgE ratio. This last parameter was preferred to specific IgE level because we would need a normalized specificity index. Finally, we included the number of dominant allergens used to calculate PRIS and decide the allergen(s) to be used with SLIT.

To our knowledge, this study is the first time that a multi-parameter score has been effective in identifying patients eligible for SLIT in a real-life setting. Overall, PRIS strongly correlates with symptom improvement after 24 months of SLIT (Figure 2), and it was effective in patients undergoing single-allergen SLIT (Figure 3A) as well as in patients treated with a two-allergen SLIT (Figure 3B). Furthermore, PRIS was a good predictor in both patients with only rhinitis (Figure 3C) and patients with rhinitis associated with asthma (Figure 3D). Together, these results indicate that PRIS can be used to predict the efficacy of SLIT regardless of the patient’s clinical condition, the product, and the number of allergens used for SLIT. Our observations suggest that PRIS also effectively predicts the degree of clinical response patients may expect from SLIT. Indeed, we observed that patients with a higher PRIS value have significantly more chances of achieving a higher symptom improvement (Figure 4). In addition, we reported that most patients with PRIS ≥ 80 experienced a very high improvement, whereas patients with 60 > PRIS ≥ 40 mostly experienced a high improvement (Figure 5). Further studies are needed to confirm these observations on a larger scale.

One of the surprising observations in our study was to find such high patient compliance to AIT. Indeed, we observed that only 10.9% of patients included in our study did not complete the 24-month follow-up. These data are dramatically lower than that reported by most studies (30–40%) [41,42,43]. We cannot exclude the possibility that the limited number of patients influenced compliance in our study. However, we believe that one of the reasons for the high patient compliance in our cohort is that the knowledge of their PRIS value conferred them a sort of awareness about the goal they could achieve with SLIT.

The clinical efficacy of AIT is measured using various scores as study endpoints. According to EAACI recommendations [37,38,39,40], we used a combined symptom and medication score (MSS) to permit the comparison of results with other studies. Our results confirmed that SLIT effectively improves symptoms of rhinitis and/or asthma after 12 months of SLIT treatment (Figure 1A). Although an additional symptom improvement was recorded after 24 months, no significant difference was observable between MSS-12 and MSS-24 (Figure 1A). Another interesting point of reflection is that up to a 30% improvement is achieved with placebo in AIT placebo-controlled studies [54]. This placebo effect is substantially less than >75% in nearly half of the patients in the present study (Table 1). However, as this study was open, there is no way to evaluate a contribution of the placebo effect on the perceived effects of the AIT. We also observed a significant response in both monosensitized and polysensitized patients (Figure 1B) and patients with or without allergic asthma (Figure 1C). In our cohort, a better efficacy was found in patients with only rhinitis than in patients with rhinitis associated with asthma (Figure 1C). However, further studies are needed to confirm this observation.

Our study is subject to some limitations. First, the sample size was small, but our encouraging results showed that a strong direct correlation between PRIS and SLIT outcome (Figure 2 and Figure 3) could be a starting point for multi-center studies, which could validate PRIS on a larger scale. Second, we used different products for SLIT, and we cannot exclude the possibility that data on SLIT efficacy can be influenced by the product used. However, we avoided any product comparison because products for SLIT cannot be compared at present due to their heterogeneous composition [55,56]. From our perspective, combining several parameters routinely used in clinical practice to obtain a disease score rather than relying on a single parameter or a single product for SLIT may help better manage the within-subject variability.

In conclusion, AIT is very demanding for the patients since it is expensive and requires a long period to achieve a sustained response [7,37]. Therefore, a specific tool able to predict SLIT efficacy is worth being used in clinical practice to select eligible patients and improve patients’ compliance to complete the course of treatment.

## Figures and Tables

**Table 1:** Classification of patients treated with sublingual immunotherapy (SLIT) based on ΔMSS. Patients were stratified based on ΔMSS to identify patients who had a better clinical response as compared to those with a poor response to SLIT. ΔMSS-12: evaluation after 12 months of SLIT treatment; ΔMSS-24: evaluation after 24 months of SLIT treatment.

	ΔMSS	ΔMSS-12 (N, %)	ΔMSS-24 (N, %)
**Very high symptom improvement**	ΔMSS ≥ 75%	39 (39.80%)	41 (41.84%)
** High symptom improvement **	50% ≤ ΔMSS < 75%	37 (37.76%)	41 (41.84%)
**Mild symptom improvement**	25% ≤ ΔMSS < 50%	10 (10.20%)	14 (14.29%)
**Low symptom improvement**	ΔMSS < 25%	12 (12.24%)	2 (2.04%)
	Total	98 (100.00%)	98 (100.00%)

**Table 2:** Predictive Response to Immunotherapy Score (PRIS).

Parameter	Group	Score	Score Range
**Age (years)**	0–12	15	3–15
	13–18	12	
	19–28	9	
	29–38	6	
	>38	3	
**Clinical features**	Rhinitis	9	3–9
	Rhinitis + Asthma	6	
	Rhinitis + Asthma + Other Allergies	3	
**Disease onset (years)**	3	9	3–9
	4–10	6	
	>10	3	
**Number of allergen sensitizations ^a^**	1	16	4–16
	2–3	12	
	4–5	8	
	>5	4	
**Presence of symptoms following exposure to allergen(s) to which** **the patient is sensitized**	Symptoms when exposed to 1 allergen	12	3–12
	Symptoms when exposed to 2 allergens	9	
	Symptoms when exposed to 3 allergens	6	
	Symptoms when exposed to ≥4 allergens	3	
**Specific IgE/total IgE** **(s/t) ratio**	s/t ≥ 0.2	12	4–12
	0.2 > s/t ≥ 0.05	8	
	s/t < 0.05	4	
**Component-Resolved Diagnosis for major allergens**	High Positive (IgE ≥ 3.50 KUA/L)	12	0–12
	Positive (0.35 ≤ IgE < 3.50)	6	
	Negative (IgE < 0.35 KUA/L)	0	
**Allergen dominance ^b^**	1	15	0–15
	2	10	
	3	5	
	>3	0	
**Total**			20–100

^a^ Assessed with Skin Prick Test and/or specific IgE; ImmunoCAP 250, Phadia, Sweden. ^b^ The number of dominant allergens was assessed as described in the Methods Section 2.5 (Immunotherapy).

**Table 3:** Allergen(s) used for sublingual immunotherapy (SLIT).

	Allergen(s)	Number of Patients N (%)
**Single-allergen** **SLIT**	Parietaria	38 (38.77%)
	House dust mite	18 (18.36%)
	Gramineae grass	18 (18.36%)
	Alternaria	1 (1.02%)
	Olive	1 (1.02%)
**Two-allergen** **SLIT**	Parietaria + Gramineae grass	27 (27.55%)
	Parietaria + mugwort	2 (2.04%)
	House dust mite + Parietaria	1 (1.02%)
	Gramineae grass + mugwort	1 (1.02%)
	Gramineae grass + olive	1 (1.02%)
	Olive + mugwort	1 (1.02%)
	Parietaria + olive	1 (1.02%)

**Table 4:** Linear regression models using as predictors all PRIS parameters.

PRIS Parameter		N	Outcome ΔMSS-24(%)		
			Beta	95% CI	*p*-Value
**Age (years)**		98			**<0.001**
*Group*	*Score*				
>38	3		—	—	
29–38	6		0.01	−0.09, 0.10	
19–28	9		0.13	0.04, 0.22	
13–18	12		0.13	0.03, 0.23	
0–12	15		0.19	0.08, 0.30	
**Clinical features**		98			**<0.001**
*Group*	*Score*				
Rhinitis + Asthma + Other Allergies	3		—	—	
Rhinitis + Asthma	6		0.12	0.02, 0.22	
Rhinitis	9		0.19	0.09, 0.29	
**Disease onset (years)**		98			**0.006**
*Group*	*Score*				
>10	3		—	—	
4–10	6		0.09	0.03, 0.16	
≤3	9		0.11	0.03, 0.18	
**Number of allergen sensitizations**		98			**0.003**
*Group*	*Score*				
>5	4		—	—	
4–5	8		−0.02	−0.12, 0.07	
2–3	12		0.05	−0.04, 0.15	
1	16		0.16	0.04, 0.29	
**Symptoms when exposed to**		98			**<0.001**
*Group*	*Score*				
3 allergens	6		—	—	
2 allergens	9		0.04	−0.04, 0.11	
1 allergen	12		0.15	0.07, 0.22	
**Specific IgE/Total IgE (s/t) ratio**		98			**<0.001**
*Group*	*Score*				
s/t < 0.05	4		—	—	
0.2 > s/t ≥ 0.05	8		0.05	−0.02, 0.13	
s/t ≥ 0.2	12		0.15	0.07, 0.22	
**CRD for major allergens**		98			**<0.001**
*Group*	*Score*				
Negative (IgE < 0.35 KUA/L)	0		—	—	
Positive (0.35 ≤ IgE < 3.50)	6		0.11	0.00, 0.21	
High Positive (IgE ≥ 3.50 KUA/L)	12		0.20	0.09, 0.31	
**Allergen Dominance**		98			**0.002**
*Group*	*Score*				
3	5		—	—	
2	10		0.02	−0.08, 0.12	
1	15		0.12	0.02, 0.22	

## Data Availability

The data presented in this study are available on request from the corresponding author.
